# Utility of live aided Cortical Evoked Potential (CAEP) for optimizing programming of cochlear implants

**DOI:** 10.1590/2317-1782/e20250106en

**Published:** 2026-03-27

**Authors:** Muthu Selvi Thangaraj, Violet Priscilla, Jeffi Johnson

**Affiliations:** 1 Department of Audiology, Sri Ramachandra Institute of Higher Education and Research, (Demmed to be University) - Chennai (Tamil Nadu), India.; 2 Sri Ramachandra Institute of Higher Education and Research (Demmed to be University) - Chennai (Tamil Nadu), India.; 3 Speech Language Pathologist – Able UK - Dubai (Abu Dhabi United Arab Emirates), UAE.

**Keywords:** Auditory Threshold, Speech Perception, Optimization, Children, Cochlear Implant

## Abstract

**Purpose:**

The present study assessed the utility of live-aided Cortical Auditory Evoked Potentials (CAEP) to optimize most comfortable level (M-levels) in children using cochlear implants.

**Methods:**

14 children using CI from both genders (5 females, 9 males) in the age range of 4 to 8 years participated in the study. A live CAEP based program was carried out by adjusting programming parameters and simultaneously recording CAEP in a free-field condition. The cochlear implant’s speech processor was connected to the programming unit. The program was switched to live mode to receive an acoustic stimulus, and simultaneously, aided CAEP was measured. The M level of the program was adjusted until the P1 peak is elicited for /b//d//g/ stimuli at 40 dBnHL. This live CAEP based program was given to the same participants for them to use for two weeks. Behavioral threshold and speech perception was measured in CEAP program and compared with participants’ everyday program.

**Results:**

Though sound field thresholds were better in live CAEP based programs, they were not statistically significant. And also, Speech perception scores were similar between every day and the live CAEP based program. Though M levels were higher in a live CAEP based program, they are not statistically significant from everyday programs.

**Conclusion:**

Live CAEP could be used successfully to optimize programming parameters. This would certainly reduce the number of trial runs and duration of programming.

## INTRODUCTION

Cochlear implants (CIs) play an important role in the rehabilitation of individuals with hearing loss who do not benefit from conventional amplification devices. A CI can be done to a child as early as 6 months of age to improve speech and language outcomes^(1)^. Children who are implanted prior to 3 years have a higher chance of matching with typically developing individuals in terms of developing linguistic skills^(2)^. As increasingly younger children undergo cochlear implantation, achieving accurate programming becomes challenging due to their inconsistent behavioral responses. Accurate programming is based on behavioural measures obtained at each electrode. This behavioural response is measured based on the determination of threshold level ("T"), most comfortable level ("M"), and loudness judgement if necessary. Not every individual can consistently demonstrate reliable behavioural responses, especially when they are young. In situations where dependable behavioural measurements are unavailable or impractical, it is essential to employ objective measures in order to determine behavioural thresholds in this population. Further, it can be used for programming cochlear implants more accurately.

Cortical Auditory Evoked Potentials (CAEPs) may be a more reliable measure for objectively fitting CIs. Given that the response is from the cortex rather than the auditory nerve, it is more likely that it corresponds with stimulus perception. CAEP can be elicited using pulse trains resembling those used in programming. CAEPs have been recorded in CI users as a measure of cortical maturation^(3)^ and as an objective measure of auditory function^(4)^.

Several studies have attempted to validate CI programming based on CAEP measures. Visram et al.^(5)^ found that electrically elicited CAEP thresholds have a high correlation with behaviorally measured T levels. This implies that an electrical CAEP threshold might be a useful programming tool to set T levels. Brown et al.^(6)^ have created three experimental programs with different acoustic and electric overlaps for bimodal users. The authors studied whether CAEP could be used to identify the best program based on the behavioural responses elicited using the speech perception task. Though there was a higher amplitude of CAEP responses in certain programs with better speech perception, it did not accurately predict the best program on the basis of speech perception scores. The author suggested that the type of stimuli used (diphthong) to measure cortical responses would have contributed to no correlation between CAEP measures and behavioural responses. The study by Martin et al.^(7)^ found changes in CAEP latency as well as auditory thresholds and speech recognition when there is a change in T/C levels in adult cochlear implant users of the Cochlear company. The author found that when the T levels increased above actual T levels, it led to improvement in sound field thresholds and P1 latency but had no effect on speech recognition performance in quiet or noise. On the other hand, when C levels were reduced below the actual C level, it worsened sound field thresholds, P1 latency, and poorer performance in speech perception in quiet and noise. Reduction of T from actual levels resulted in poor sound field threshold and delayed P1 latency. However, Thangaraj et al.^(8)^ reported different results for cochlear company users in children. The authors found that CAEP responses, as well as sound field threshold and speech perception, worsened only with reduced C levels. The above studies imply that CAEP responses change with modification of C level. Using a similar concept, Távora-Vieira et al.^(9)^ used CAEP to optimize the CI fitting in 14 adult CI users with post-lingual single-sided deafness (SSD). The authors have constructed the program by adjusting Most comfortable level (MCL levels until there are clear CAEP responses for all speech tokens of different frequencies presented. All participants adapted to the CAEP based program. The authors found that CAEP can be used to objectively verify programming for adult CI users with SSD.

Children with cochlear implants should have an aided threshold of 20 to 30 dB HL to hear distant whispers and soft speech^(10)^. Aided CAEP threshold is obtained 15 dB above the behavioural threshold^(11,12)^. The traditional approach for validation of programming involves measuring behavioural threshold and speech perception after programming. Adjusting programming parameters based on behavioral responses requires numerous trial runs. In order to construct a CAEP based program, the current study adjusted the programming parameters while simultaneously recording CAEP responses. Additionally, the behavioural responses were used to validate the CAEP based program. Thus, the present study attempted to find the utility of live CAEP to optimize CI programming in children using cochlear implants. The study had two objectives. First, to compare the behavioral aided sound field threshold and speech perception between everyday and live CAEP based programs. Second, to compare the “M” levels between everyday and live CAEP optimized programs.

## METHOD

### Ethical consideration

The study was approved by the institutional Ethics Committee (Reference number CSP/21/SEP/99/471) of Sri Ramachandra Institute of Higher education and Research (Deemed to be university). Written consent was taken from parents prior to data collection.

### Participants

14 children from both genders (5 females, 9 males) using CI, with an age range of 6 to 9 years (mean age of 7.5 years) participated in the study. All participants used an AB device of HiRes90K 1j electrodes. The mean age of implantation is 3.2 years, and the duration of implant use is 4.2 years. All participants were diagnosed with congenital severe to profound hearing loss. Participants had a minimum of one year of hearing experience with the cochlear implant. All of them had complete insertion of the electrode array and uneventful surgery. All participants had normal inner ear and auditory nerve anatomy based on Computed Tomography (CT) and Magnetic Resonance Imaging (MRI) reports. None of the children had any additional disabilities related to cognition, Auditory Neuropathy spectrum disorder (ANSD), autism, or hyperactivity disorder. Participants who had absent CAEP responses due to various factors such as poor family support or poor compliance to the aural rehabilitation therapy were excluded from the study. The participant’s details are given in Table 1.

**Table 1:** Details of participants

**Name**	**Age (Year)**	**Gender**	**Age of Implantation (Year)**	**Duration of use (Year)**	**Implanted ear**	**Implant model**
**1**	7.2	Male	3.1	4.1	Right	HiRes 90K1j with Naida Q- 70
**2**	6.8	Male	4.6	2.2	Right	HiRes 90K1j with Harmony
**3**	5.4	Female	3.2	2.2	Right	HiRes 90K1j with Harmony
**4**	8.3	Male	3.4	4.9	Right	HiRes 90K1j with Naida Q30
**5**	7.6	Female	4.8	2.8	Right	HiRes 90K1j with Naida Q30
**6**	7.4	Male	3.4	4	Left	HiRes 90K1j with Naida Q30
**7**	8.7	Female	3.0	5.7	Right	HiRes 90K1j with Naida Q30
**8**	8.4	Male	4.7	3.7	Left	HiRes 90K1j with Harmony
**9**	8.2	Male	1.6	6.6	Left	HiRes 90K1j with Naida Q- 70
**10**	8.6	Male	3.8	4.8	Right	HiRes 90K1j with Harmony
**11**	7.1	Male	2.1	5	Right	HiRes 90K1j with Harmony
**12**	7.3	Female	1.4	5.9	Left	HiRes 90K1j with Harmony
**13**	6.7	Male	3.2	3.5	Right	HiRes 90K1j with Harmony
**14**	7.4	Female	3.1	4.3	Left	HiRes 90K1j with Naida Q30
**Mean**	7.5		3.2	4.2		

### Equipment

A calibrated diagnostic audiometer Grason-Stadler (GSI) 61-SN (AU1CE15102671) was used to record sound field thresholds for frequency-modulated tones. The aided CAEPs with a cochlear implant were measured using a two-channel Duet IHS (SN:00822034) instrument. Preloaded speech stimuli of /b/, /g/, and /d/ of IHS with different spectrums of low, mid and high frequency respectively were included in the study.

### Material for speech perception

A picture identification test in Tamil was used to assess speech perception in children using cochlear implants^(13)^. The picture identification test, which has four choices, consists of bisyllabic words with corresponding pictures in Tamil. This test comprises two equalized lists, each with 25 words. This test can be administered to children with normal hearing and cochlear implant users between the ages of 3 and 6 years.

**Everyday program:** Since variability is present in programming the speech processor, the procedure to set the T and M levels is briefly explained here. The recommendation by Wolfe and Schafer^(10)^ was followed while setting the M level of everyday program. The software used to create the everyday program for AB users was Sound Wave version 3.1. Speech coding strategy of HiRes S Optima with APWII mode were used as default setting to create everyday program. The M levels were set using four sets of speech bursts from electrodes 1–16. On a three-point loudness rating scale, participants assessed the loudness of speech bursts presented in ascending order. The M level for specific electrodes was set as the highest level that was the most comfortable level to the participants. Next, the levels of the remaining electrodes were interpolated. The M levels were globally lowered and evaluated in live mode. T levels were established at 10% of M levels in accordance with AB's programming recommendations. The Ling 6 sound test was used at a distance of six feet to confirm speech perception and loudness tolerance. Participants used this program for a minimum of 6 months.
**Procedure:** Live Cortical Auditory Evoked Potential-based optimized programming was carried out in two steps. Step 1: Creation of a base program; Step 2: Fine-tuning of the base program based on a live CAEP recording.
***Creation of base program***: Participant‘s speech processor was connected to a programming system. A base program was created based on objective measures of threshold of Electrically Evoked Compound Action Potentials (ECAP) at selected five electrodes for each participant (on 1,4,8,12,16^th^ electrodes). The selected electrodes were evenly spaced from base to apical electrodes. The base program was created using the same default processing strategy, stimulation rate, and pulse width mentioned in the everyday program. M levels were kept at the threshold of ECAP, and the remaining electrodes are interpolated in programming software. it was ensured that these levels were comfortable for participants by individually sweeping across the electrodes. In addition, they were presented with loud drum, bell sounds in a live voice to ensure that it is comfortable.
***Fine-tune the program based on a live CAEP recording****:* Initially, a speech processor with the base program was switched on in live mode, and simultaneously, CAEP was recorded. The following paragraph explains fine-tuning based on the live CAEP recording.

The participant was shown age-appropriate, silent cartoon films. The child psychologist provided approval to these animated films, which contained no violent elements. preloaded speech stimuli of /b/, /g/, and /d/ with low, mid, and high frequency spectra, respectively, were chosen to evaluate aided benefit across frequencies. The speech stimuli had durations of 114 ms, 206 ms, and 213 ms. These stimuli were presented at a rate of 1.1 s via a speaker at varying intensities, ranging from 80 dB nHL to 40 dB nHL.

CAEP responses were recorded on one channel, and ocular artefacts were observed on the other. Eye blinks were removed using a bipolar electrode montage (lateral outer canthus referenced to superior orbit). Whenever the participants blinked their eye, artefact rejection was activated to remove the eye blink artefact online. To prevent radio frequency (RF) artefacts, the inverting electrode is placed on the non-implanted mastoid (A1 or A2), and the non-inverting electrode of the first channel is placed on the vertex (Cz). A common electrode was attached to the lower forehead (Fpz). It was ensured that the inter-electrode impedance was less than 2 kΩ and the absolute electrode impedance was less than 3 kΩ before recording. Both the response and the recorded EEG were amplified 25,000 times. A bandpass filter ranging from 1 to 30 Hz was used to filter the amplified responses. Responses for 150 sweeps were averaged. To reduce the muscle artefact, the artefact rejection was set at 50 µV. Using a 500-ms time window with a 100-ms pre-stimulus averaging window, recorded responses were displayed. Each recording was done twice to verify the replicability.

The goal was to record CAEP at 40dBnHL for the specified stimuli. The intensity was set in accordance with Wolfe and Schafer's recommendations^(10)^. The authors suggested that an aided threshold of 20 to 30 dB HL should be obtained to verify the audibility after CI programming. The behavioural threshold is typically 10–15 dB higher than the CAEP threshold^(14,15)^. Then, CAEP has to be present at 40 dB nHL to validate the programming. This was the reason that programming levels were adjusted until the CAEP response for the stimuli /b/, /d/, and /g/ was detected at 40 dB nHL for the present study. There were two reasons why only M levels were used for fine-tunning in the present study. First, only M level is adjusted during programming and T is automatically adjusted to 10% of M levels, as per AB device programming requirements. Furthermore, based on previous research, only the Maximum current level changes lead to changes in CAEP and behavioural measures^(7,8)^.

**Figure 1:**
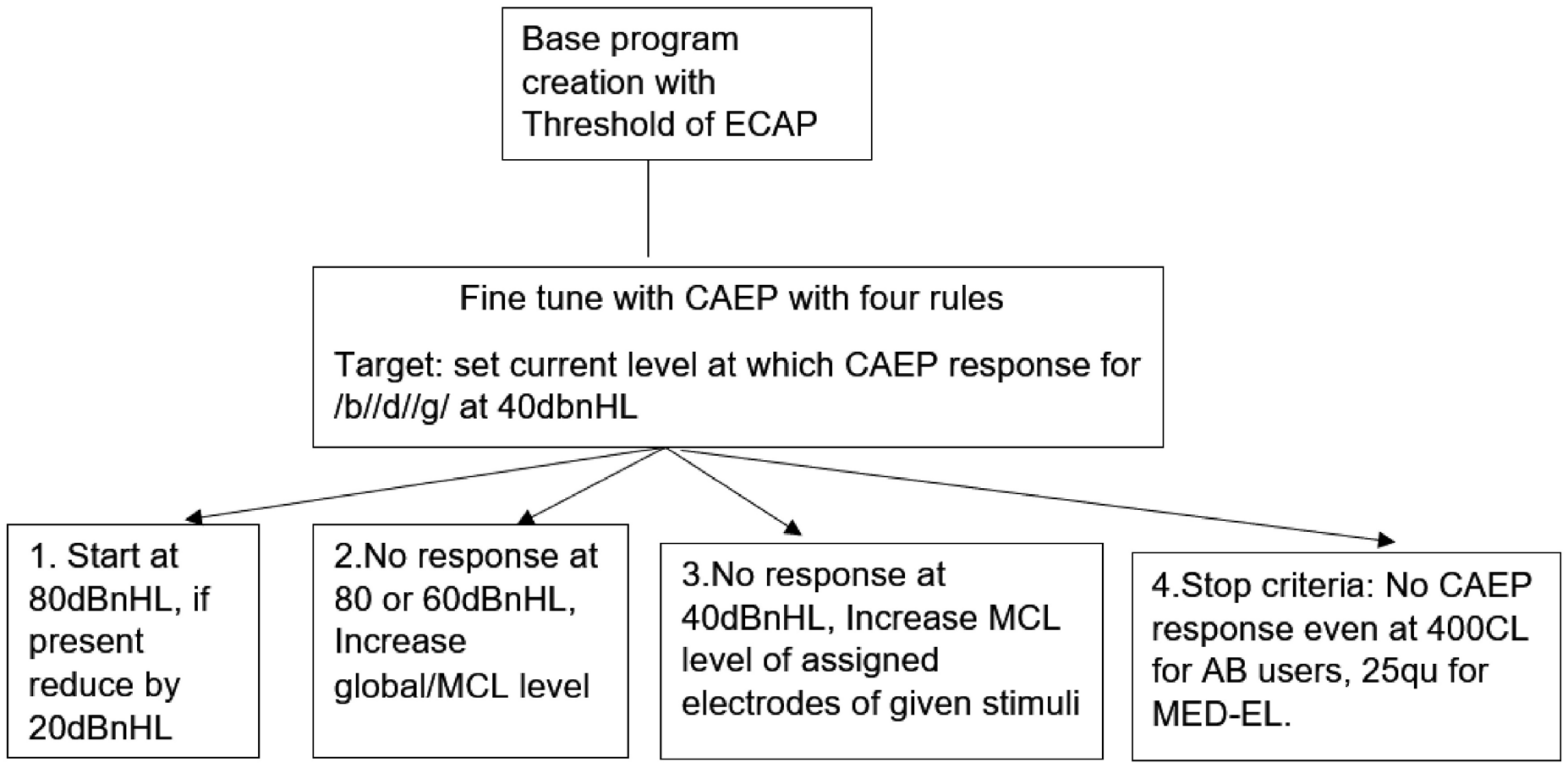
Schematic representation of depicting fine tuning procedure for CAEP based program

Four rules were used to fine-tune the programming based on CAEP (Figure 1). First, testing was always started at 80 dBnHL, and if a response was detected at this level, the intensity was decreased by 20 dB. Second, the M level was raised globally if neither 80 dB nHL nor 60 dB nHL elicited any response for any of the stimuli of /b/, /d/, & /g/. Third, if there was no response at 40 dBnHL for any specific stimuli, the M level of the assigned electrodes of stimuli frequencies was raised. Fourth, there was a stop criterion that current levels were never increased beyond 400 CL for AB users though there was no response obtained. This was done to avoid overstimulating the participant with uncomfortable, loud stimuli.

**Figure 2:**
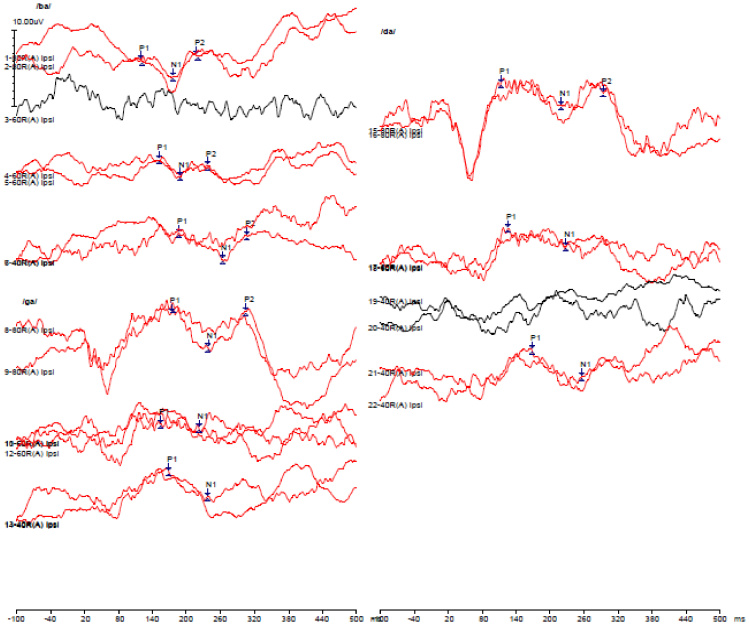
Shows P1 obtained at 40 dB nHL for /ba/ /da/ /ga/ in the final CAEP based program. Wave program in black indicates CAEP waveform prior to fine tuning

The following paragraph and Figure 2 explain the fine-tuning process of a CAEP based program for one participant. If CAEP was recorded at 80 dBnHL for the stimuli /b/ for the base program. Overall M levels were increased in smaller steps. Current levels were increased until there was a response at 40 dB nHL for stimuli /b/. Similarly, if CAEP responses were not observed for high-frequency stimuli of /d/ at 40 dBnHL. Overall M levels at basal electrodes were adjusted until there was a response at 40 dBnHL. These current levels were smoothed or interpolated to create the final program. In addition, it was ensured that these levels were comfortable for the participants. Thus, a CAEP based program is the final program with P1 that was obtained for the lowest stimulation level at 40 dBnHL for stimuli of /b/, /d/, and /g/. Figure 2 depicts the CAEP waveform obtained pre- and post-fine-tuning, also the waveform at 40dBnHL in the final program.

The participants were required to use the CAEP-based program for two weeks. if the CAEP-based program caused participants any discomfort. This program was provided in a progressive format to use at home. Behavioural indicators of speech perception and aided threshold were examined in both everyday programs and CAEP-based programs.

**Sound field aided thresholds:** Participants were seated comfortably at a 1-meter distance from speakers at a 45° angle towards the test ear. Warble tones of different frequencies from 250 Hz to 8 kHz were presented through speakers from calibrated GSI 61 audiometer in the free-field room. Minimum intensity at which the participants perceived was considered as sound field thresholds. Sound field threshold was found out using the Hughson-Westlake procedure^(16)^.
**Speech perception measures:** speech perception was done using a picture identification test in Tamil for children^(13)^. The participant was seated comfortably at a 1-meter distance from the speaker with a 45-degree azimuth towards the test ear during the testing. Recorded lists were delivered through speakers at 50 dB nHL at 10 dB singal to noise ratio (SNR)in the presence of speech noise. The participant was instructed to point at the stimulus image of a word heard. The total number of correct answers for each participant was converted into a percentage score. The testing of subtests was done in a random order.

During the pilot study, most participants achieved a ceiling score of 90–100% in a quiet environment. Due to these reasons, the testing was conducted at the 10 dB SNR level for every day and CAEP based program. Speech noise was presented at 40 dBHL with reference to the speech signal at 50 dBHL. The authors could not obtain an open-set word perception score due to the presence of misarticulation in 50% of the population.

CAEP based program was done by the author. Different examiners collected behavioral assessments of aided thresholds and speech perception for everyday and CAEP based program. The examiners who examined behavioral measures had no knowledge of the program's setting or the outcomes of cortical auditory assessments in each program.

### Statistical analysis

Statistical analysis was carried out using Statistical Package for Social Sciences (SPSS), version 26. As data did not follow the normal distribution curve as per the Shapiro-Wilk test, the median of the sound field threshold, speech perception, and M levels in both the everyday and CAEP based programs were found using the 50th quartile value. Wilcoxon signed tests were done to compare sound field threshold, speech perception, and M levels between everyday and CAEP based program.

## RESULTS

### I. Comparison of sound field threshold and Speech Perception Scores between everyday and CAEP based program

**Figure 3:**
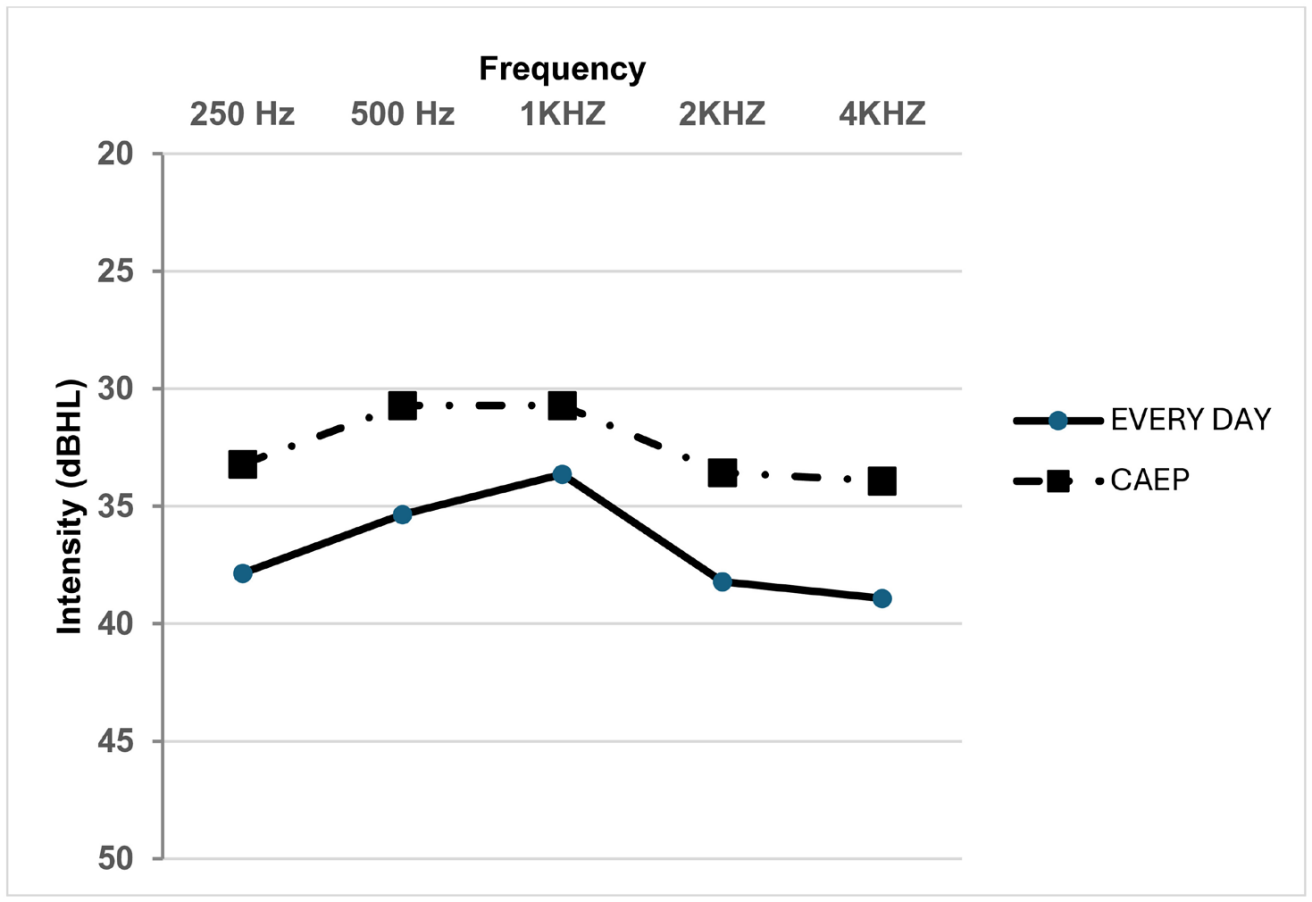
Median sound field thresholds between everyday and CAEP-based program (N=14)

**Figure 4:**
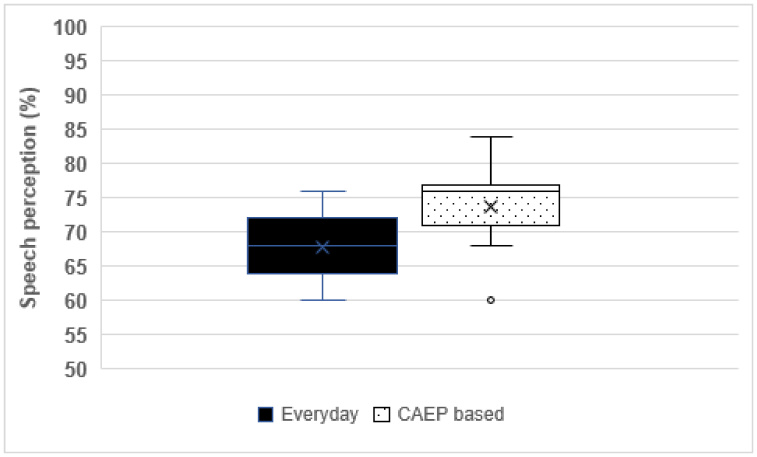
Box plot showing median, quartile of speech perception at 10dBSNR between the everyday program and the CAEP-based program

There was an improved median sound field threshold across frequencies from 250 Hz to 8 kHz in the CAEP based program compared to the everyday program (Figure 3). However, according to the Wilcoxon signed-rank test, the sound field threshold across frequencies was not significant (p > 0.05) between every day and the CAEP based sound program. Speech perception was higher in the CAEP based program than in the everyday program (Figure 4). Yet, there was no significant difference between the everyday and the CAEP based program in terms of speech perception scores (z = 0.00, p = 1) based on the Wilcoxon signed-rank test. As per the above results, it was interpreted that both the sound field threshold and speech perception measured between the everyday and the CAEP based program are similar.

### II. Comparison of the M levels between everyday and CAEP based program in individuals using MED-EL and Advanced Bionics

**Figure 5:**
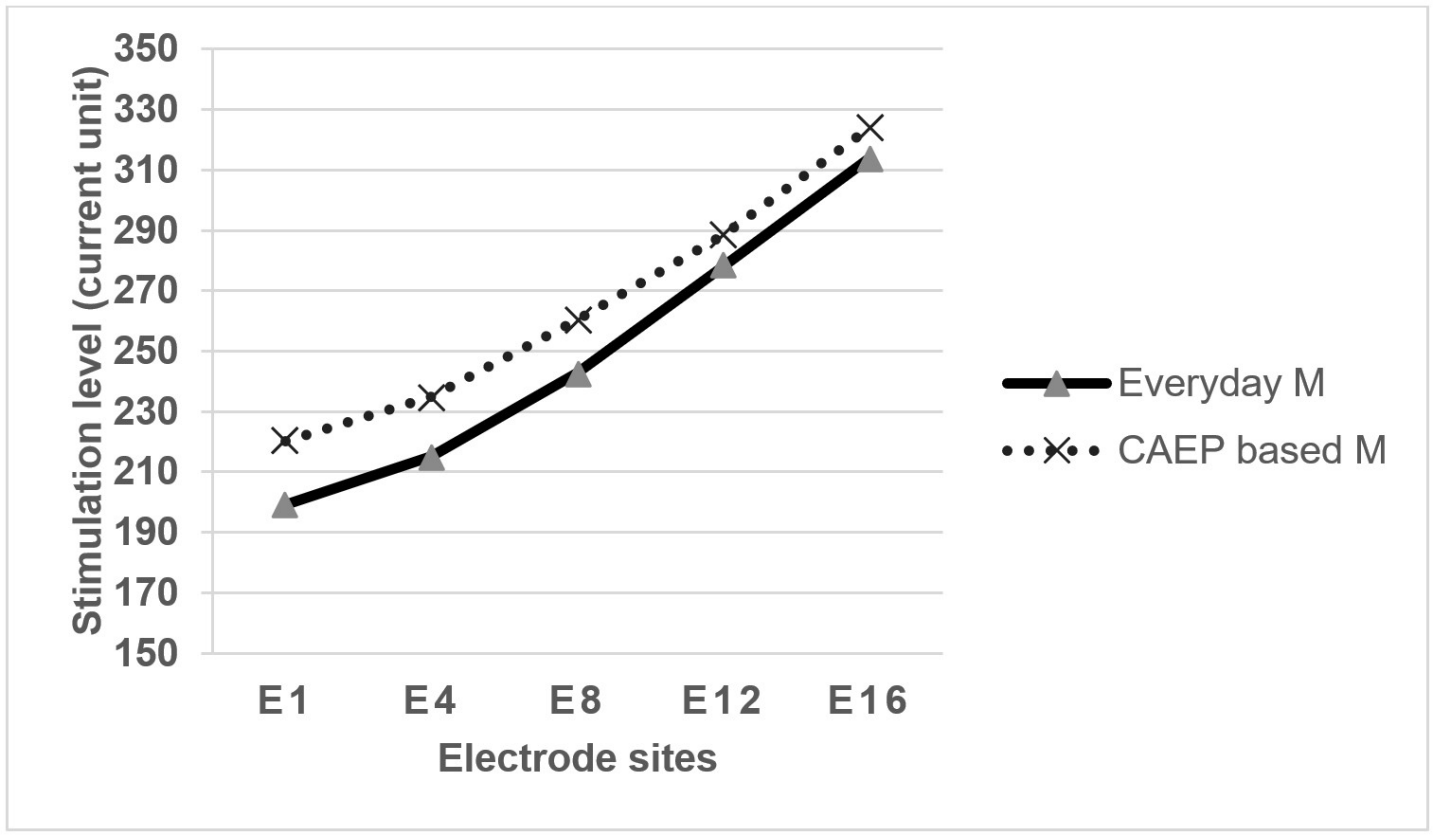
Median T/M levels in everyday program and CAEP-based program for AB users (N=14)

There was a higher median M level in the CAEP based program compared to the everyday-based program for AB users (Figure 5). However, according to the Wilcoxon signed test, there was no significant difference (p = 0.08) between the everyday and the CAEP based sound program in terms of M levels in AB users. Based on the above results, it was interpreted that the M levels measured between the everyday program and the CAEP based program were similar.

## DISCUSSION

The results indicated that the aided thresholds and speech perception in the CAEP based program had improved audibility and speech perception compared to the everyday program. However, they were not statistically significant. Two different equalised word lists were used to measure speech perception for CAEP based and everyday program in the present study. Even though the CAEP based program resulted in improved audibility, this effect was not noticed in speech perception. This can be attributed to the fact that speech perception was measured at a higher level of 50 dBHL (around 20 dBSL). Brown et al.^(6)^ also found that though there was an improved amplitude of CAEP in certain program that had higher speech perception, these differences were not statistically significant.

Similar to the present study, Tavora Vieira et al.^(9)^ described how CAEP could be used to optimize CI programming. For those who did not achieve desired CAEP responses at certain frequencies of speech tokens at all intensities, similar to the present study, their M levels were increased until there was a response observed at all intensities for all speech tokens. Though some of them initially did not accept the program, as they felt it was loud and high-pitched in their study, they got acclimatised over time and had improved audibility and clarity. Similarly, in the present study, some children (n = 3) felt that the CAEP based program was loud; however, they got acclimatised over time when behavioural testing was carried out after two weeks.

Overall, median T/M levels in the CAEP based program were higher compared to the everyday program across the electrodes. However, these levels were not statistically significant. Martins et al.^(7)^ also observed improvements in sound field threshold and speech perception with increase in T level. An increase in T levels in CAEP based program levels would also have resulted in an improved sound field threshold (around 5dB). Martins et al.^(7)^ and Thangaraj et al.^(8)^ also found out that a reduction in C level resulted in reduced audibility and speech perception in cochlear users. And also, Thangaraj et al.^(8)^, found out that there was a reduction in P1 amplitude and an increase in CAEP threshold and P1 latency when C levels were lower than the actual level.

As CI programming is validated based on audibility and speech perception, And also, results on these measures are comparable to the everyday program. It can be concluded that a live CAEP based program could be a good option to optimize the programming. Previous investigations have also highlighted the significance of CAEP in evaluating speech perception and the objectivity of audibility assessments. Ching et al.^(17)^ supported the idea that CAEP can be used to validate the audibility of hearing aid users. Similarly, several investigators have used CAEP to verify audibility in young children as well as in hearing aid users^(17,18)^. And also, several studies have found a favorable correlation between speech perception and CAEP findings^(18,19)^.

## CONCLUSION

There were no statistical differences in M level, sound field threshold, or speech perception elicited between the everyday and CAEP based program. Thus, Live CAEP could be successfully used to optimize CI programming. This would certainly reduce the number of trial runs and their duration while programming. However, the clinician might incorporate CAEP with appropriate caution in clinical practice for the following reasons: The present can be directly incorporated for only AB users. where only M levels are adjusted. In contrast, adjusting T levels is essential for cochlear users. Further studies can be done to see how live CAEP can be utilized in that scenario. As CAEP morphology improves only with stimulation after implantation over time^(3)^, CAEP based programming might be difficult in the initial months of CI activation due to poor morphology.
